# Metabolomics of Thrips Resistance in Pepper (*Capsicum* spp.) Reveals Monomer and Dimer Acyclic Diterpene Glycosides as Potential Chemical Defenses

**DOI:** 10.1007/s10886-019-01074-4

**Published:** 2019-06-08

**Authors:** Mirka Macel, Isabella G. S. Visschers, Janny L. Peters, Iris F. Kappers, Ric C. H. de Vos, Nicole M. van Dam

**Affiliations:** 1 Molecular Interaction Ecology, Institute of Water and Wetland Research (IWWR), Radboud University, P.O. Box 9010, 6500 GL Nijmegen, The Netherlands; 2 Molecular Plant Physiology, Institute of Water and Wetland Research (IWWR), Radboud University, P. O. Box 9010, 6500 GL Nijmegen, The Netherlands; 3 Laboratory of Plant Physiology, Wageningen University and Research, P.O. Box 658, 6700 AR Wageningen, The Netherlands; 4 Wageningen Plant Research, Bioscience, Wageningen University and Research, P.O. Box 16, 6700 AA Wageningen, The Netherlands; 5 German Centre for Integrative Biodiversity Research (iDiv) Halle-Jena-Leipzig, Deutscher Platz 5e, 04103 Leipzig, Germany; 6 Institute of Biodiversity, Friedrich Schiller University Jena, Dornburger-Str. 159, 07743 Jena, Germany

**Keywords:** Capsianosides, *Frankliniella occidentalis*, Liquid chromatography-mass spectrometry, Insects, Solanaceae, Thrips

## Abstract

The development of pesticide resistance in insects and recent bans on pesticides call for the identification of natural sources of resistance in crops. Here, we used natural variation in pepper (*Capsicum* spp.) resistance combined with an untargeted metabolomics approach to detect secondary metabolites related to thrips (*Frankliniella occidentalis*) resistance. Using leaf disc choice assays, we tested 11 *Capsicum* accessions of *C. annuum* and *C. chinense* in both vegetative and flowering stages for thrips resistance. Metabolites in the leaves of these 11 accessions were analyzed using LC-MS based untargeted metabolomics. The choice assays showed significant differences among the accessions in thrips feeding damage. The level of resistance depended on plant developmental stage. Metabolomics analyses showed differences in metabolomes among the *Capsicum* species and plant developmental stages. Moreover, metabolomic profiles of resistant and susceptible accessions differed. Monomer and dimer acyclic diterpene glycosides (capsianosides) were pinpointed as metabolites that were related to thrips resistance. Sucrose and malonylated flavone glycosides were related to susceptibility. To our knowledge, this is the first time that dimer capsianosides of pepper have been linked to insect resistance. Our results show the potential of untargeted metabolomics as a tool for discovering metabolites that are important in plant – insect interactions.

**Electronic supplementary material:**

The online version of this article (10.1007/s10886-019-01074-4) contains supplementary material, which is available to authorized users.

## Introduction

Plant resistance to insects is often reduced in crops compared to their wild ancestors, due to breeding efforts to minimize the unwanted taste or toxic effects of some of the plants’ natural chemical defenses. Modern varieties of cabbage (*Brassica oleraceae*) for example, have very low glucosinolate levels, typical anti-herbivore defenses in the Brassicaceae family (Gols et al. 2008). To grow healthy crops, application of insecticides has therefore become a necessity. However, not all insects can be controlled effectively by insecticides, partly due to a recent increase in insecticide resistant insect populations (Bass and Jones 2018). Moreover, sustainable agricultural practices call for finding natural insect resistance in crops to reduce the need for insecticides that are harmful to the environment, *e.g*. neonicotinoids (Hallmann et al. 2014). Because genetically modified crops are largely banned from Europe, novel sources of resistance preferably come from wild crop relatives that can be used in breeding schemes.

*Capsicum* species (Solanaceae) are grown world-wide as economically important crops, such as hot and sweet peppers, which are mostly *Capsicum annuum* L*.* and *Capsicum chinense* L.. A major pest on *Capsicum* are thrips, an order of tiny insects that can cause severe damage (Steenbergen et al. 2018). In greenhouses in Western Europe, western flower thrips (*Frankliniella occidentalis*) is the prevalent pest species (Kirk and Terry 2003). *F. occidentalis* is notoriously difficult to control with insecticides, due to its thigmotactic behavior and fast evolving insecticide resistance (Bielza 2008). There are a few studies on insect resistance in *Capsicum* (*e.g*. Fery and Schalk 1991; Maharijaya et al. 2011, 2012; Mollema and Cole 1996; Yang et al. 2011)*,* but the mechanisms underlying insect resistance in this genus still remain largely unknown. Next to physical defenses such as hairs, almost all plants produce chemical defenses. In this study we aim to identify the chemical defenses of *Capsicum* species by using the natural variation in thrips resistance among *Capsicum* accessions.

Plants produce a wealth of primary and secondary metabolites. Plant chemical defenses are mostly secondary metabolites (Schoonhoven et al. 2005). The estimated number of secondary metabolites in the plant kingdom is 200.000 (Weckwerth 2003). Plant secondary chemistry is often highly species specific, with some metabolites only occurring in a single species or genus (Macel et al. 2014; Schweiger et al. 2014). Studies on plant chemical defenses have mostly focused on well-known plant secondary metabolites such as alkaloids or glucosinolates. However, these known chemical defenses are only a fraction of all plant secondary metabolites. Hence, bioactive metabolites may be overlooked when using targeted analytical approaches. Novel analytical techniques and advanced bioinformatics make it possible to detect and identify an unprecedented number of metabolites. Using untargeted metabolomics analyses, many metabolites of different compound classes can be detected in a single analysis (Macel et al. 2010). The benefit is that next to the ‘knowns’ also yet unknown metabolites can be detected, which are related to plant resistance to herbivores or pathogens (Leiss et al. 2009). The downside of untargeted metabolomics is that some of the unknowns will remain unknown because annotation of the detected metabolites is still a daunting task (Peters et al. 2018).

Here, we used an untargeted metabolomics screening approach in combination with thrips feeding assays to understand the mechanisms of insect resistance in *Capsicum*. The chemistry of *Capsicum* fruits is well-studied, mostly in relation to food quality and flavor (Kim et al. 2017; Martín et al. 2017; Wahyuni et al. 2014). Capsaicinoids in the fruits, alkaloids that give the hot peppers their pungent taste, are known to have anti-microbial activity (Tewksbury et al. 2008). Chemistry the leaves is less well investigated, but several studies indicate that secondary plant metabolites of *Capsicum* play a role in pathogen and insect resistance. For example, the terpenoid capsidiol plays a role in induced defenses against pathogens (Lee et al. 2017). Volatiles emitted by *C. annuum* plants can attract the Chilli thrips *Scirtothrips dorsalis* (Shivaramu et al. 2017). High levels of tocopherols and low levels of aromatic amino acids in leaves of *Capsicum* have been related to thrips resistance (Maharijaya et al. 2012; Mollema and Cole 1996). In addition, a recent study on metabolite QTLs and thrips resistance using a *C. annuum* x *C. chinense* F2 mapping population showed that high levels some acyclic diterpene glycosides were weakly but significantly correlated to thrips mortality (Maharijaya et al. 2018).

We selected 11 *Capsicum* accessions from a group of 40 accessions that were screened for resistance to the western flower thrips *F. occidentalis* (Visschers et al. 2019). These 11 accessions, seven *C. annuum* and four *C. chinense*, were classified either resistant or susceptible to *F. occidentalis*. In this study, the same 11 accessions were tested again for thrips resistance using leaf disc feeding choice-assays. Because insect resistance may be plant age dependent (Barton and Koricheva 2010; Cipollini and Redman 1999; Visschers et al. 2019), we tested leaves from plants in the vegetative stage as well as leaves from flowering plants in the feeding choice assays. Leaves of these plants were also analyzed with untargeted LC-MS metabolomics. Multivariate analyses were used to link the metabolomic profiles with the thrips feeding damage in order to pinpoint metabolites related to thrips resistance or susceptibility in *Capsicum*.

## Material and Methods

### *Capsicum* Accessions and Plant Growth Conditions

Eleven *Capsicum* accessions were selected from a thrips resistance screening of 40 accessions. These eleven accessions were selected for being either most or least resistant (Visschers et al. 2019). Original seed material was provided by the Center for Genetic Resources (CGN) of Wageningen University and Research Centre, The Netherlands (http://cgngenis.wur.nl/) (Table S1). Seeds were multiplied by selfing three plants per accession in the greenhouse, which were combined in a bulk seed lot per accession. F1 seeds were germinated on sterile glass beads with demi water in a growth cabinet (Snijders DeLuxe, Tilburg, The Netherlands) at 25/20 °C, 16/8 L/D, 70% humidity. F1 seeds were germinated in closed plastic cups (7 cm diameter) on sterile glass beats (1 mm diameter) and demi water in a growth cabinet at 30/20 °C, 16/8 L/D, 70% humidity. When the first two true leaves had developed, the seedlings were transplanted to plastic pots (11 cm × 11 cm × 12 cm) containing commercial potting soil. The pots were placed in a greenhouse, inside an insect-free net cage at 16/8 L/D and minimum temperatures set to 20 °C/17 °C (day/night). Natural light was supplemented with Greenpower lights. Plant were given nutrient solution once a week (1.8% Kristalon Label Blue, Yara, Grimsby, UK). For each accession 8 to 16 plants were grown and included in the metabolomics analysis. A subset of the plants (8 to 10) of each accession were also used in the thrips choice assays.

### Thrips Choice Leaf Disc Assay

After 4 weeks of growth, when all plants were still in the vegetative stage, one fully grown leaf from the upper part per plant was used for thrips feeding assessment. Per accession 8–10 plants were used for this choice experiment. Leaf disc experiments were performed as described by Visschers et al. (2018a). Briefly, using a cork borer, two leaf discs (1.5 cm diameter) were punched from each leaf, thereby avoiding the mid-vein. A leaf disc from each accession was placed on a drop of 1.5% slightly liquid agar with the abaxial side up in a Petri dish (9 cm diameter). Each Petri dish (*n* = 20) thus contained 11 leaf discs (placed in a circle), each representing 1 of the 11 accessions. The order of the leaf discs was randomized for each replicate, the first disc of each set was indicated with a star underneath the Petri dish. Ten Petri dishes were inoculated with thrips, the other ten were used as controls. Per inoculated Petri dish, 22 L1/L2 *F. occidentalis* larvae, reared on green beans and starved for 24 h prior to experiments, were placed in the middle of the dish using a small painting brush. All Petri dishes were sealed with Parafilm and placed in the same climate cabinet as used for insect rearing. Petri dishes without thrips were directly sealed with Parafilm and used for correction during image analysis. After 48 h thrips were removed, and thrips damage was assessed using imaging software (Visschers et al. 2018b). After 17 weeks of plant growth, when all plants were flowering, the thrips leaf disc choice assay was repeated, again using leaves from the upper part of the plant.

### LC-MS Sample Preparation and Metabolite Extraction

At the same time when leaves were harvested for the choice assay, one leaf of the same age per plant was harvested for the untargeted LC-MS based metabolomics profiling. Per accession, 8–16 individual plants were used for the chemical analyses. Directly upon harvesting, leaves were flash frozen in liquid nitrogen, freeze dried and stored at −80 °C. Freeze dried material was finely ground with a Ball Mill (Retsch MM 300) and 20 mg per sample was used for metabolite extraction for LC-MS. A pool sample was made by combining equal amounts of freeze dried material from all accessions and both developmental stages; this pooled sample was treated similarly and simultaneously as the experimental samples and used as a quality control. Samples were extracted with 1 ml 75% methanol +0.1% formic acid, sonicated at room temperature at 40 kHz for 15 min and then centrifuged for 10 min at 15000 rpm (De Vos et al. 2007). A 500 μl aliquot of each sample was transferred into a 96-well LC-MS plate.

### LC-MS(MS) Analyses

Samples were put in randomized block order for LC-MS analyses. Every 20 samples an extract from the sample pool was placed as a quality control. LC-MS analyses were performed as described previously (Mokochinski et al. 2018). In short, separation of compounds in the crude extracts was performed using an HPLC system (Waters Acquity, Milford, USA) generating a flow rate of 0.19 mL.min^−1^ and a 45 min gradient of 5 to 75% acetonitrile in water, acidified with 0.1% formic acid, on a C_18_ reversed phase column (Luna 150 × 2 mm i.d., 3 μm; Phenomenex, Torrance, USA) kept at 40 °C. Detection of eluting compounds was by a PDA detector (Waters) at 210–600 nm and subsequently an LTQ-Orbitrap FTMS hybrid mass spectrometer (Thermo Scientific, Bremen, Germany). Samples were analyzed in both positive and negative ionization modes. A mass resolution of 60,000 FWHM was employed during data acquisition in a mass range of *m/z* 90–1350. Additional LC-MS/MS analyses on selected samples were performed (van der Hooft et al. 2012).

### LC-MS – Data Preprocessing

Baseline correction and peak-wise alignment was done using Metalign (Lommen 2009). The threshold for signal to noise ratio was set at 3. Data were filtered by removing peaks that were present in less than 8 samples over the entire dataset. For further multivariate data analyses, the peak amplitudes were normalized to 10,000 total peak amplitude for each sample (relative abundance of peaks compared to the total peak amplitude).

### LC-MS Mass Feature Clustering

Cluster analyses were run in MSClust to group those mass peaks that likely belong to the same metabolite, based on their corresponding retention times and relative abundance across samples (Tikunov et al. 2012). This procedure was repeated with remaining mass peaks not assigned to a cluster in the first cluster analysis, after which the two cluster analyses were combined. The cluster analyses of the negative mode data resulted in 1966 clusters, or so-called reconstructed metabolites, to which roughly half of the peaks could be assigned. The mass clustering was used to identify parent ions and facilitate annotation of the metabolites. The relative abundance of metabolites was expressed in MIC values (*i.e.* Measured Ion Counts) which was used as parameter for the multivariate analyses.

### Metabolite Annotation

Metabolite annotation was based on manually checking the putative molecular ion within the clustered mass signals of selected metabolites. Metabolites were then annotated using an *in-house* database (Wageningen Plant Research - Bioscience, the Netherlands) based on comparisons of retention time, accurate mass, isotopic composition, UV spectra and MS/MS information, as well as on-line available metabolite databases such as KNApSAcK, KEGG and MassBank. Putative acyclic diterpene glycosides detected in negative mode were confirmed by MS analysis in positive mode based on the presence of their common fragment with specific accurate m/z 271.2424 (Heiling et al. 2016). In our untargeted metabolomics analyses, we detected double charged fragments that were correlated with thrips resistance. These fragments likely belonged to diterpene glycosides, dimer capsianosides, with a molecular weight higher than our initial *m/z* range. For confirmation of the dimer capsianosides, the LC-MS/MS mass range was extended to *m/z* 150–2000 and selected samples of accession RU 63 were analyzed in both positive and negative ionization modes. Dimeric capsianosides have an *m/z* > 1400, monomers have an *m/z* < 1400 (KNApSAcK).

### Statistical Data Analyses

Data analyses were performed in R 3.5.0 (www.r-project.org) and Simca 15 (Umetrics, Umea, Sweden). Thrips choice assay data were calculated as mm^2^ eaten per leaf disc / total amount of mm^2^ eaten per petri dish × 100 (relative amount eaten per leaf disc in a petri dish) for each leaf disc. These data were analyzed with a Friedman ANOVA for dependent data. Post-hoc pairwise differences in thrips damage between accessions were analyzed with paired Wilcoxon signed ranks tests. Based on the choice assays results, accessions were classified as either resistant (< 9% damage; if no preference among 11 choices, 9% damage is expected on each disc) or susceptible (> 9% damage). This resistance classification was used in the multivariate analyses.

To investigate the overall chemical diversity of the accessions, the total number of detected mass signals among the accessions and developmental stages were analyzed with a two factorial ANOVA with accession and developmental stage as fixed factors. Within each developmental stage, the difference in detected mass signals between susceptible and resistant accessions was also analyzed with ANOVA, with resistance grouping as fixed factor and accession nested in resistance grouping.

To investigate differences in metabolomic profiles, both the mass signal dataset as well as the cluster dataset (MIC) were used for multivariate analyses and subsequent relevant metabolite selection (peak picking). The mass clustering software is still under development, by using both datasets we achieved the most complete and reliable screening of metabolites. Focus of the data analyses was on the negative MS ionisation mode, which was more consistent in peak detection over time, whereas the positive ionization mode data were mostly used for the annotation of the metabolites. Metabolomic profiles were first explored with unbiased Principal Component Analyses (PCA). Further analyses for identifying metabolites underlying resistance or susceptibility were done using Partial Least Square – Discriminant Analyses (PLS-DA). In these supervised PLS-DA models, the resistance classification of each accession was included in the model. All plants were used in the metabolomics screening, and a subset was also used for the thrips bio-assays. For the set of plants of which we obtained both the metabolomics data as well as thrips damage estimates, we also performed Partial Least Square Regression analyses where % damage data was regressed against the full metabolomic profiles. The PLS(−DA) models were cross-validated with permutation tests (999 permutations). Q^2^ values of all permuted models were compared to the Q^2^ values of the real data. Q^2^ values of the real data should be higher than 0.5, and all permuted Q^2^ should be lower than the real Q^2^. Significance of the PLS models was also tested with cross-validated ANOVA (Eriksson et al. 2008; Triba et al. 2015). Subsequently, the PLS loading plots and variable influence on the projection (VIP) loadings of significant models were used to make an initial selection of clusters/mass signal of interest (VIP > 2). To confirm our PLS(−DA) results, relative abundance of the clusters/mass signals from this initial cluster/mass selection were correlated with % thrips damage at individual plant level (Spearman rank correlations). Those masses/clusters that showed a significant or near significant correlation with thrips damage, either positive or negative, were selected for metabolite annotation.

## Results

### Thrips Feeding Preference

**Fig. 1:**
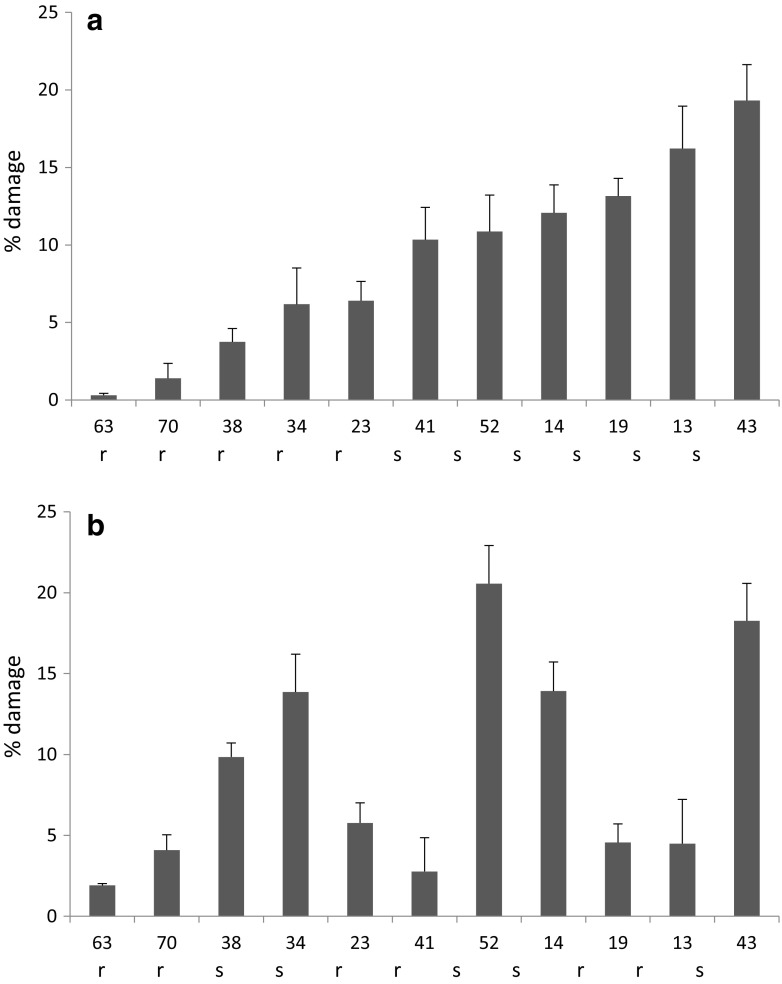
Mean percentage *Frankliniella occidentalis* leaf damage (+SE) per accession in choice tests with leaf discs of 11 *Capsicum accessions***a**) when plants were 4 weeks old (vegetative) or **b**) flowering (generative). X-axis labels indicate RU seedbank accession numbers (see Table 1). Accessions are ordered based on feeding preference in the vegetative stage. Overall differences between accessions are significant in both vegetative (A) and flowering (B) stage (both P < 0.0001,*P*- values are based on Friedman ANOVAs for dependent data, for pairwise differences between accessions see Table S2). The letters r and s indicate classification of the accession as either resistant (r < 9% damage) or susceptible (s > 9% damage). *N* = 8–10 per accession

The thrips leaf disc choice assay showed significant differences in feeding damage among the 11 accessions in both the vegetative stage and the generative stage (Fig. 1, Friedman ANOVA, vegetative stage χ^2^ = 57.6, *df* = 10, *P* < 0.0001; generative stage χ^2^ = 38.6, *df* = 10, *P* < 0.0001; see Table S2 for pairwise differences between the accessions). In our leaf disc assay, feeding was lowest on accession RU 63 and high on RU 43 in both the vegetative stage and generative stage (Fig. 1). For some accessions, thrips preference depended on plant developmental stage. For example, RU 34 received relatively little damage in the vegetative stage, but became more susceptible in the generative stage. Similarly, RU 52 became more susceptible when flowering. In contrast, RU 13 and RU 19 were susceptible in the vegetative stage but became more resistant when the plants where flowering (Fig. 1).

### Metabolomic Profiling – Mass Signals Detected

After data filtering, the negative ionization mode yielded 24,531 detected mass signals and the positive ionization mode 52,530 mass signals over all samples. The number of total detected masses in the negative mode differed significantly among accessions (ANOVA, *df* = 10, *F* = 40.6, *P* < 0.001).

In some accessions there was a significant increase in number of detected masses in the generative stage compared to the vegetative stage (accession x developmental stage: *F* = 13.42, *P* < 0.001, Table 1). This was most pronounced in RU 13 (Table 1), which also was more resistant in the generative than in the vegetative stage (Fig. 1). In the generative stage, resistant accessions had on average significantly more detected masses than susceptible accessions (Table 1, ANOVA, *df* = 1, *F* = 11.36, *P* = 0.008). In the vegetative stage, there was no difference in number of detected masses between resistant and susceptible accessions (Table 1, ANOVA, *df* = 1, *F* = 1.24, *P* = 0.292) .

**Table 1:** Radboud University (RU) codes of *Capsicum* accessions, species, and the average number of detected mass peaks (±SE) in leaves per accession at the vegetative or generative developmental stage in the negative ionization mode of the MS are indicated. *P* values of Tukey post-hoc tests after ANOVA indicate differences between developmental stages. Resistance column indicates whether an accession was classified as resistant (r) or susceptible (s) based on the choice assay results from Fig. 1. Note that this may change from the vegetative stage to the generative stage. *N* = 8–16 per accession

RU no.	Species	Detected mass peaks				*P*
		Vegetative	Resistance	Generative	Resistance	
63	*C. annuum*	6561 (±87)	r	7237 (±77)	r	<0.0001
23	*C. annuum*	6852 (±81)	r	7099 (±66)	r	0.065
19	*C. annuum*	6616 (±119)	s	7171 (±75)	r	<0.0001
34	*C. annuum*	6507 (±109)	r	5948 (±122)	s	<0.0001
43	*C. annuum*	6066 (±146)	s	5943 (±134)	s	0.363
52	*C. annuum*	5673 (±107)	s	5697 (±196)	s	0.241
14	*C. annuum*	6143 (±78)	s	7052 (±132)	s	<0.0001
38	*C. chinense*	5753 (±91)	r	5939 (±65)	s	0.194
41	*C. chinense*	5591 (±44)	s	6633 (±65)	r	<0.0001
70	*C. chinense*	5746 (±96)	r	6444 (±101)	r	<0.0001
13	*C. chinense*	5771 (±55)	s	7013 (±141)	r	<0.0001
Average	*Resistant*	6341 (±67)		7012 (±50)		
	*Susceptible*	6041 (±61)		6077 (±82)		

### Metabolomic Profiling – Multivariate Analyses

**Fig. 2:**
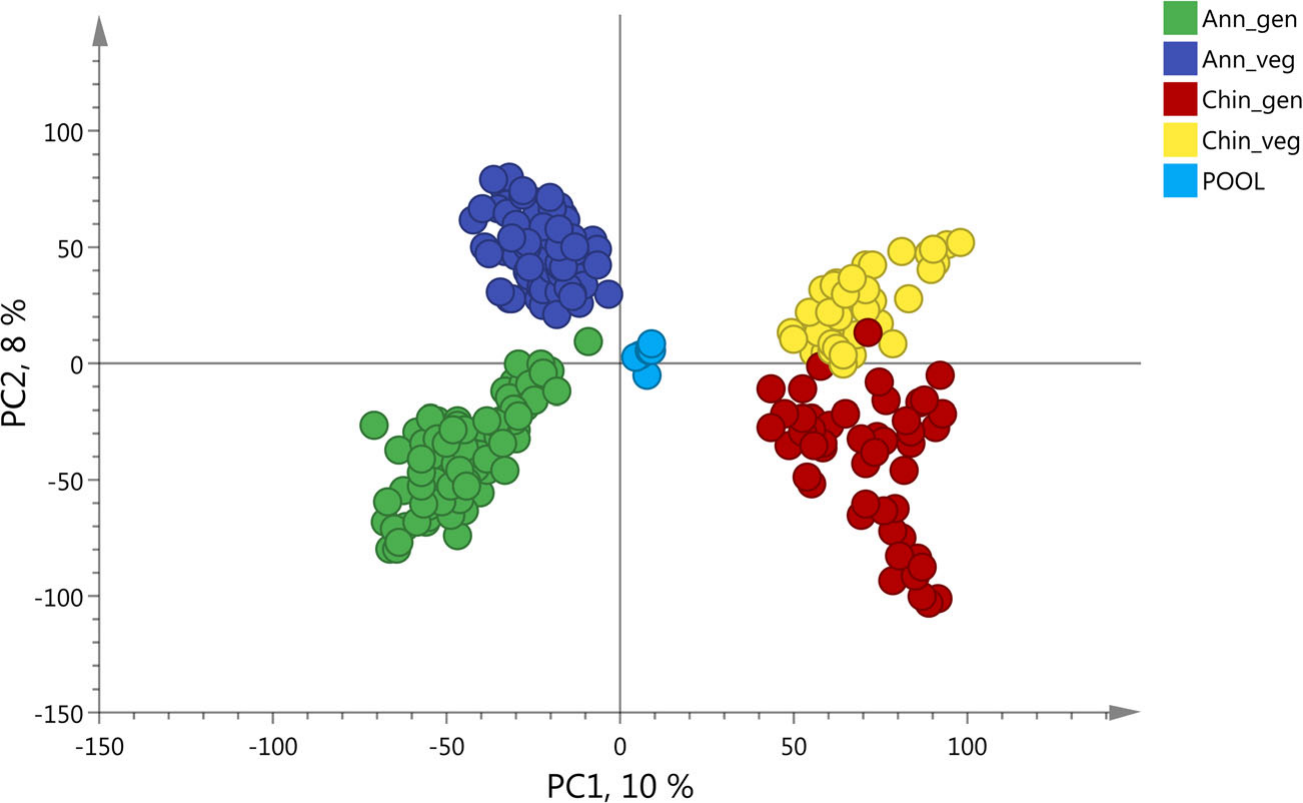
Principal Component Analyses (PCA) of LC-MS untargeted metabolomics analyses of *Capsicum* leaves in negative ionization mode. Different colors indicate different species and developmental stages. Ann_veg: *C. annuum* vegetative stage (dark blue dots), Ann_gen: *C. annuum* generative stage (green dots), Chin_veg: *C. chinense* vegetative stage (yellow dots), Chin_gen: *C. chinense* generative stage (red dots). Light blue dots indicate the pool samples

**Fig. 3:**
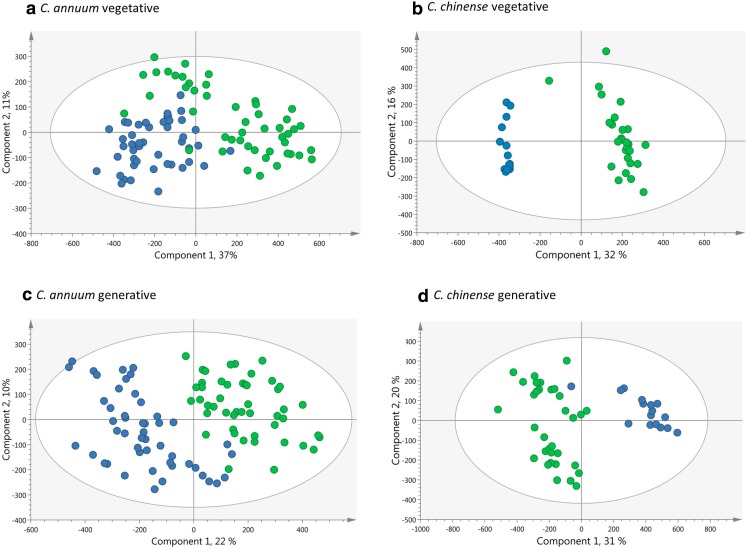
Partial Least Square- Discriminant Analysis (PLS-DA) plots of LC-MS mass clusters (MIC) per *Capsicum* species and developmental stage. **a**) *C. annuum* vegetative stage, **b**) *C. annuum* generative stage, **c**) *C. chinense* vegetative stage, **d**) *C. chinense* generative stage. Green dots indicate plants of resistant accessions, blue dots indicate plants of susceptible accessions. All models were cross-validated with permutation tests (Q^2^ > 0.75, *P* < 0.0001). *N* = 8–16 per accession

**Fig. 4:**
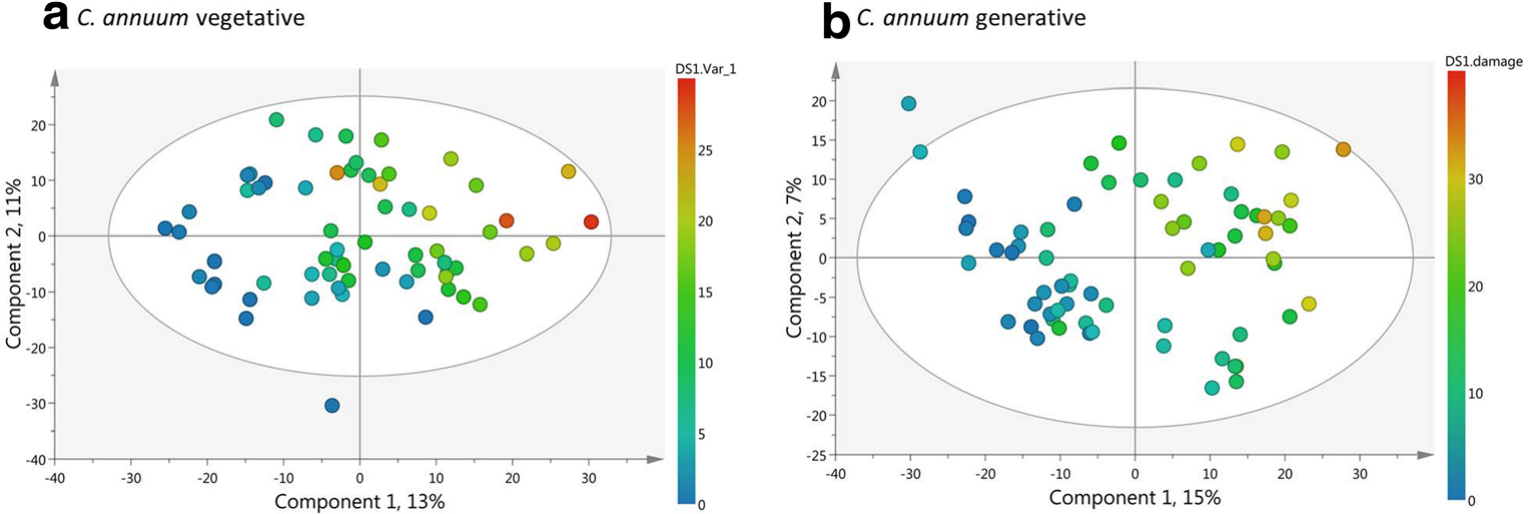
Partial Least Square regression plots of *Capsicum annuum* LC-MS metabolomic profiles (MIC) and % thrips damage. **a**) plants in vegetative stage, **b**) generative stage. More blue colors indicate less thrips damage. Both models were significant (Q^2^ > 0.6, *P* < 0.005). *N* = 6–10 per accession

The PCA of all data in both negative and positive ionization modes showed a clear separation of the metabolomics profiles based on species (PC 1) and developmental stage (PC 2) (Fig. 2, Fig. S1). Based on these results, further analyses in search for resistance factors in the metabolomics dataset were performed per species and developmental stage separately. Firstly, unsupervised PCA analyses of each species and developmental stage already showed a grouping based on resistance class (Fig. S2) and on accessions (Fig. S3). Subsequent supervised PLS-DAs which incorporated the resistance classification of each accession in the models, showed a clear separation of metabolomic profiles based on resistance class (Fig. 3, Fig. S4). Cross-validation permutation tests of the PLS-DA models showed that all models were significant and not overfitting (CV-ANOVA all *P* < 0.001; all Q^2^ > 0.75, all Q^2^ permuted models < 0.3). For those plants of which we obtained both the metabolomics data as well as thrips damage estimates, we also linked thrips preference to metabolomic profiles by using PLS regression models. These PLS regression models of *C. annuum* were significant and showed a separation of metabolomics profiles based on thrips damage levels (Fig. 4, Fig. S5). For *C. chinense* these PLS regression models were not significant (*P* > 0.7, Q^2^ < 0.2), likely due to smaller sample size for this species, and were thus not used for further analyses.

### Metabolite Identification

**Fig. 5:**
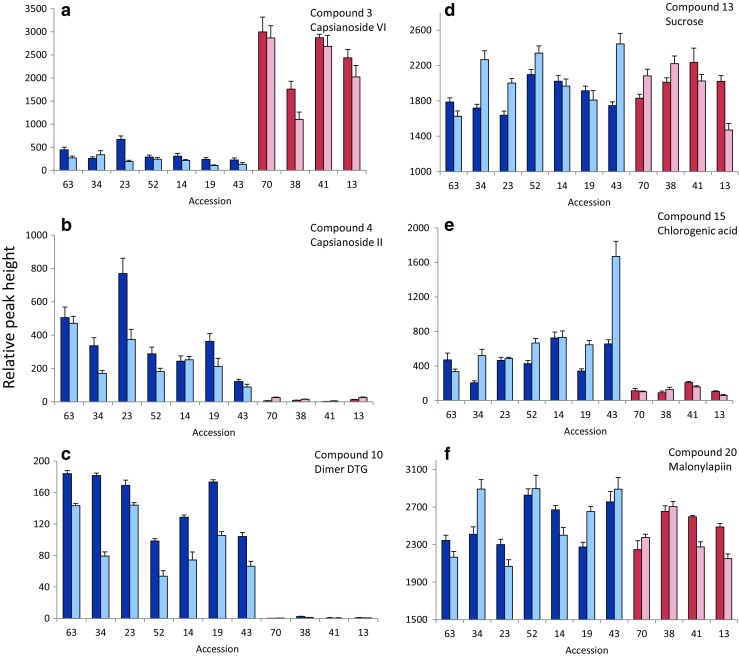
Examples of relative abundance of peak heights of selected metabolites per *Capsicum* accession (+SE) in the vegetative stage (dark bars) and the flowering stage (light bars). *C. annuum* accessions are indicated in blue, *C. chinense* accessions in red. Compound numbers correspond with those in Table 2. **a**) 3. Capsianoside VI, **b**) 4. Capsianoside II, **c**) 11. Dimer Capsianoside, **d**) 13. Sucrose, **e**) 15. Chlorogenic acid, **f**) 20. Malonylapiin. See Fig. S6 for relative abundances of all metabolites from Table 2

The PLS-DA loading plots and PLS regression loading plots were used to select clusters/masses that contributed most to the differences among groups and damage levels. Spearman correlations further showed (near) significant correlations between relative abundance of part of these masses and percentage thrips damage (Table 2). Annotation of these masses based on MS(MS) fragmentation patterns revealed that the metabolites that were related to resistance were mostly capsianosides, a group of acyclic diterpene glycosides (DTG) (Table 2). The relative abundance of both monomer capsianosides and dimer capsianosides were significantly negatively correlated with thrips preference (Table 2). While Capsianoside II was more abundant in *C. annuum* compared to *C. chinense*, Capsisanoside VI was more abundant in *C. chinese*, indicating species-specific capsianoside profiles (Fig. 5ab, Fig. S6). Capsianoside VI emerged from the *C. chinense* PLS-DA as related to resistant accessions, but was nevertheless poorly directly correlated with thrips preference (Table 2). The dimer capsianosides related to thrips resistance were mostly only detected here in *C. annuum* (Fig. 5c, Fig. S6, Fig. S7). Furthermore, two flavone glycosides were related to resistance (compound 1 and 2), although other flavone glycosides (compounds 18–22) were related to susceptibility (Table 2). These flavone glycosides related to thrips susceptibility mostly had a malonyl group (compound 17–18 and 19–20), such as malonylapiin (Table 2). Chlorogenic acid and sucrose were also positively correlated with thrips damage (Table 2). The relative abundance of sucrose and in particular chlorogenic acid was high in leaves of thrips susceptible accession RU 43 when plants were flowering (Fig. 5de). Furthermore, sucrose levels in RU 13 dropped in the flowering stage, while they increase in RU 52 and RU 34 (Fig. 5d). This pattern matches with RU 13 becoming more resistant and RU 52 and RU 34 becoming more susceptible in the flowering stage compared to the vegetative stage (Fig. 1).

**Table 2:** Relevant metabolites in *Capsicum* spp. related thrips (*Frankliniella occidentalis*) resistance/susceptibility. Annotation based on MS(MS) fragmentation patterns in both positive and negative ionization mode. Annotation of capsianosides was based on presence of 271.24 aglycon fragment in positive ionization mode. m/z: mass-to-charge ratio, Rt: retention time in minutes, R_s_: Spearman Rank Correlation Coefficient of correlation between relative peak abundance and % thrips damage per species/developmental stage. Small letters after R_s_ indicate species/stage(s) in which a correlation(s) was found; ^av^) *C. annuum* vegetative, ^ag^) *C. annuum* generative stage, ^cv^) *C. chinense* vegetative stage, ^cg^) *C. chinense* generative stage. Lowest R_s_ and *P* value are given in case a metabolite was pinpointed in multiple analyses

Compound no.	*m/z* MH-	Rt	Molecular formula	Annotation	R_s_
1	563.1407	14.9	C_26_H_28_O_14_	Apigenin-O-glycoside	−0.35^+cv^
2	679.1543	17.3	C_30_H_32_O_18_	Luteolin methyl ether	−0.37^+cv^
3	921.4693	23.6	C_44_H_74_O_20_	Capsianoside VI	−0.17^cg^
4	1083.5238	21.7	C_50_H_84_O_25_	Capsianoside II	−0.39**^av,ag^
5	1169.5247	22.5	C_53_H_86_O_28_	Monomer Capsianoside	−0.52***^av,ag^
6	1169.5248	23.0	C_53_H_86_O_28_	Monomer Capsianoside	−0.50***^av^
7	1185.5165	20.9	C_53_H_86_O_29_	Capsianoside II + malonyl	−0.50***^ag^
8	1735.7971	27.5	C_82_H_128_O_39_	Dimer Capsianoside	−0.72***^ag^
9	1563.7969	28.0	C_76_H_124_O_33_	Dimer Capsianoside	−0.39*^av,ag^
10	1649.7977	28.2	C_79_H_126_O_36_	Dimer Capsianoside	−0.70***^av,ag^
11	1735.7976	28.7	C_82_H_128_O_39_	Dimer Capsianoside	−0.77***^ag^
12	1735.7971	29.5	C_82_H_128_O_39_	Dimer Capsianoside	−0.76***^av,ag^
13	341.1089	2.1	C_12_H_22_O_11_	Sucrose	0.50***^ag^
14	343.1074	21.0	?	Unknown	0.70***^cv^
15	353.0876	8.7	C_16_H_18_O_9_	Chlorogenic acid	0.46***^ag^
16	371.2065	14.3	C_13_H_22_O_2_ hexoside	Unknown	0.40**^av,ag^
17	473.1093	17.7	C_24_H_21_O_13_	Apigenin-O-malonyl hexoside	0.60***^ag^
18	533.0937	15.9	C_24_H_22_O_14_	Luteolin-O-malonyl hexoside	0.50***^ag,cv^
19	581.1529	9.2	C_26_H_30_O_15_	Naringenin-C-hexoside-pentoside	0.56***^av^
20	649.1407	16.9	C_29_H_30_O_17_	Malonylapiin	0.45***^av,ag^
21	665.1389	15.4	C_29_H_30_O_18_	Luteolin-diglucoside` malonate	0.83***^cv,av^

+ *P* < 0.10

*P ≤ 0.01

**P ≤ 0.001

***P ≤ 0.0001

## Discussion

Our untargeted metabolomics analyses in combination with thrips feeding assays of multiple *Capsicum* accessions revealed metabolites that were related to thrips resistance and susceptibility. Several monomer and dimer acyclic diterpene glycosides (DTG) were related to resistance, while sucrose, flavone glycosides and chlorogenic acid were related to susceptibility. To our knowledge, this is the first study that links the dimer DTG to thrips resistance. Our results are in line with other recent studies on DTG and highlight the emerging role of DTG as defenses against insects in Solanaceae (Heiling et al. 2010; Poreddy et al. 2015; Maharijaya et al. 2018).

The thrips choice assays showed significant differences in thrips feeding among the selected *Capsicum* accessions. Within the selected accessions, RU 63 was the most resistant, which is consistent with earlier studies (Maharijaya et al. 2011) that included this and other accessions. RU 43 and RU 52 were the most susceptible. Interestingly, three accessions became more resistant when they started flowering, while two other accessions became more susceptible. Age-related resistance has been shown in disease resistance (*e.g.* Kus et al. 2002) and has also been described for resistance to insects (*e.g.* Beck 1964; Cipollini and Redman 1999; Stout et al. 2013). Generally, it is thought that changes in intrinsic factors, such as resource acquisition and allocation, as well as changes in extrinsic factors, such as herbivore selection pressures, drive the evolution of age-related resistance (Barton and Boege 2017). The chemical mechanisms behind age-related resistance of our *Capsicum* accessions remain unclear, although our data suggest this could be related to shifts in relative abundance of sucrose in the leaves.

*C. annuum* and *C. chinense* had species-specific metabolome profiles. Nevertheless, within each species and developmental stage, there were overall differences in metabolomic profiles between thrips resistant and susceptible *Capsicum* accessions. Metabolites that were negatively correlated with thrips damage were annotated as acyclic diterpene glycosides (DTG), so-called capsianosides in *Capsicum* species. There are two types of DTG in *Capsicum*; monomer capsianosides I-XVIII with either 17-hydroxygeranyllinalool (HGL), 13-hydroxygeranyllinalool-16-oic acid aglycone or geranyllinalool-16-oic acid as aglycone, and dimer capsianosides A-L, with HGL and 13-hydroxygeranyllinalool-16-oic acid or geranyllinalool-16-oic acid as aglycones (Izumitani et al. 1990; Yahara et al. 1991; Lee et al. 2008). A recent mQTL (metabolite QTL) study linked four monomer capsianosides from *Capsicum* with thrips survival (Maharijaya et al. 2018). Our metabolomics analyses revealed that also dimer capsianosides are related to thrips resistance. We detected ten capsianosides that were related to thrips resistance, five of which were dimers. Interestingly, of these ten capsianosides only compound #7 (Capsianoside II + malonyl) was also found in the mQTL study of Maharijaya et al. (2018). Capsianosides may also have antimicrobial activity (Bacon et al. 2017). Similar monomer 17-hydroxygeranyllinalool diterpene glycosides (HGL-DTG) in *Nicotiana* species are acting as direct defenses against multiple insect herbivores (Snook et al. 1997; Heiling et al. 2010). The 17-hydroxygeranyllinalool diterpene backbone is thought to be the active component of the DTG (Snook et al. 1997). The sugars and malonyl groups that are attached to this terpene backbone are proposed to enable transport and storage inside the plant and prevent autotoxicity (Heiling et al. 2010).

Our data suggest species-specific abundance of particular capsianosides. For example, capsianoside VI was more abundant in *C. chinense* than in *C. annuum,* and capsianoside II was more abundant in *C. annuum.* Differential activity of modifying enzymes such as glycosyltransferases may lead to differences in capsianosides profiles among *Capsicum* accessions and species (Wahyuni et al. 2014), as was shown in *Nicotiana* (Heiling et al. 2016). A major QTL for thrips resistance co-localized with two DTG (Maharijaya et al. 2018). The capsianosides are also present in *Capsicum* fruits (Wahyuni et al. 2013), and it is unclear how high levels may affect taste or quality of the fruits, although they may have dietary benefit (De Marino et al. 2006). Interestingly, in fruits the mQTLs for capsianosides were localized at different chromosomes than the mQTLs of some capsianosides present in the leaves (Maharijaya et al. 2018; Wahyuni et al. 2014). We studied the constitutive chemical defenses of *Capsicum*, but it seems likely that the capsianosides also play a role in herbivore induced defenses. DTG synthesis in *N. attenuata* is induced upon herbivore feeding, which is regulated by the jasmonate signaling pathway (Heiling et al. 2010). Elucidating the molecular pathways and genes involved in capsianoside production, induction and diversity may shed light on the genetic mechanisms of DTG-based insect resistance in *Capsicum*.

At least 18 monomer and 12 dimer capsianosides have been described thus far in *Capsicum* species (Lee et al. 2008; Yahara et al. 1991,). In our additional MS/MS analysis of selected samples of *C. annuum* RU 63 we detected 6 monomer and 12 dimer capsianosides (Fig. S7). Not all of these emerged from our untargeted metabolomics study as linked to thrips resistance. It is possible that not all capsianosides are equally effective against *F. occidentalis.* Bioactivity of DTG may depend on their glycosylation pattern (Poreddy et al. 2015), but this needs further research with targeted analyses specifically aimed at detecting both monomer and dimer capsianosides. Furthermore, due to limitations of our MS-based analytical platform, mainly the inability to assign exact sugar positions and linkages to these complex molecules (Heiling et al. 2016), detailed annotation of the dimer capsianosides was not possible.

Several flavone glycosides (compound 17–21) were positively correlated with thrips damage, although two other flavones were correlated with resistance in *C. chinense* (compound 1–2). Structurally related metabolites can differ in their effects on insects. For the flavones for example, luteolin-D-glycoside from *C. annuum* was not deterrent to the leaf miner *Lyriomyza trifolli,* while the structurally related luteolin-apiosyl-glucoside was highly deterrent (Kashiwagi et al. 2005). Luteolin itself was toxic to thrips (Leiss et al. 2013). Here, we found that an apigenin-O-glycoside and a luteolin methyl ether were related to thrips resistance, but that some malonylated luteolin and apigenin glycosides were related to susceptibility to thrips. The role of malonylation in defense needs further study (Heiling et al. 2010).

Sucrose was related to thrips susceptibility, which is not surprising because thrips need the sugars from the plant to survive and grow (Nielsen et al. 2010). Chlorogenic acid was also related to susceptibility but has been related to insect resistance in other plant species (*e.g.* Leiss et al. 2009; Dillon et al. 2017). In our study, chlorogenic acid was particularly abundant in one of our most susceptible *C. annuum* accessions in the flowering stage (RU 43). Our results showed that chlorogenic acid per se is not an effective defense against thrips in *Capsicum*. Possibly, differences in polyphenol oxidase activity (enzymes involved in oxidative activation of phenolic compounds) among species determine the role of chlorogenic acid in plant – insect interactions (Appel 1993). Moreover, the effectivity of plant compounds is likely determined by the background metabolome in which they are present, which is different for each species and genotype. In a similar way, gene function may alter depending on the background genome (Chandler et al. 2013). Artificial diet experiments with alkaloids and chlorogenic acid showed that indeed the chemical background influenced the bioactivity of the combination of these compounds on thrips survival (Liu et al. 2018).

In a similar way, interactions between capsianosides and other compounds of the metabolomes of different accessions may alter the effect of capsianosides on resistance. Maharijaya et al. (2018) also suggest that several metabolites acting in concert may be responsible for thrips resistance in *Capsicum*. In our study, there was no clear trend that capsianoside abundance went up in those accessions that became more resistant in the flowering stage. Possibly, it is the combination of capsianosides and sucrose or other compounds that are important for resistance (*e.g.* high levels of capsianosides, low levels of sucrose). Metabolite richness or diversity of the metabolome in itself may further play a role in insect resistance (Macel et al. 2014). In our *Capsicum* study, resistant accessions had on average significantly higher chemical diversity than susceptible accessions in the flowering stage, although this was not significant in the vegetative stage. Compounds can act synergistically and a wider range of metabolites is thought to act as defenses against a wider range of attackers (Berenbaum et al. 1991).

Untargeted metabolomics provides a more comprehensive insight into the plant metabolome and can be used as a tool to screen for the unknowns that are important in plant – insect interactions. Furthermore, interactions between compounds cannot be revealed with targeted chemical analytical approaches. Once interesting metabolites have been identified, fine-tuned targeted chemical analytical procedures and additional experiments with gene-silenced plants and/or bioassays with pure compounds could further elucidate their effect on insects. The group of acyclic diterpene glycosides that we pinpointed in our pepper study have previously been related to broad spectrum insect resistance in *Nicotiana* species. Within the Solanaceae, DTG have been detected in the genus *Capsicum* spp., *Lycium barbarum* and several *Nicotiana* species, but were absent from Tomato (*Solanum lycopersicum*) and Potato (*Solanum tuberosum*) (Heiling et al. 2016). Similar DTG were found in Asteraceae (Akter et al. 2016). These are all fairly recent studies; it is thus possible that DTG occur in other plant species belonging to different plant families. Increasing the levels of DTG in elite breeding lines could be a way to increase insect resistance in pepper crops, provided that there is no trade-off with yield or food quality. Using natural variation of insect resistance in wild relatives of crops in combination with novel chemical and genetic technologies can lead to the discovery of new sources of insect resistance that may enhance crop protection.

## Electronic supplementary material

ESM 1 (PDF 591 kb)

ESM 2 (PDF 1409 kb)

ESM 3 (XLSX 90 kb)
