# Supplementary material for: Metabolomics of Thrips Resistance in Pepper (Capsicum spp.) Reveals Monomer and Dimer Acyclic Diterpene Glycosides as Potential Chemical Defenses
Source: J Chem Ecol. 2019 Jun 8;45(5):490–501. doi: 10.1007/s10886-019-01074-4 (PMC6570690; doi:10.1007/s10886-019-01074-4)
Supplement: Supplementary file 1 — (PDF 591 kb) [file 10886_2019_1074_MOESM1_ESM.pdf]

Electronic Supplementary Material 1

Macel *et al*. Natural variation in insect resistance of pepper (Capsicum spp.) reveals monomer and dimer acyclic diterpene glycosides as potential chemical defenses.

Table S1. *Capsicum* source material used from the Centre for Genetic Resources, the Netherlands (CGN); Radboud University (RU) codes, species and CGN numbers.

RU#	*Capsicum* species	CGN number
63	*C. annuum*	23765
23	C. annuum	22151
19	C. annuum	16913
34	C. annuum	23222
43	*C. annuum*	17227
52	*C. annuum*	21550
14	*C. annuum*	23289
38	*C. chinense*	17004
41	*C. chinense*	16994
70	*C. chinense*	16995
13	*C. chinense*	21557
