# Supplementary material for: Metabolomics of Thrips Resistance in Pepper (Capsicum spp.) Reveals Monomer and Dimer Acyclic Diterpene Glycosides as Potential Chemical Defenses
Source: J Chem Ecol. 2019 Jun 8;45(5):490–501. doi: 10.1007/s10886-019-01074-4 (PMC6570690; doi:10.1007/s10886-019-01074-4)
Supplement: Supplementary file 2 — (PDF 1409 kb) [file 10886_2019_1074_MOESM2_ESM.pdf]

Electronic Supplementary Material 2

Macel *et al*. Natural variation in insect resistance of pepper (*Capsicum* spp.) reveals monomer and dimer acyclic diterpene glycosides as potential chemical defenses

Table S2. Paired Wilcoxon signed-rank tests results (V-statistics, *P*-value) on relative leaf disc damage by *F. occidentalis* on the different *Capsicum* accessions (RU numbers). Bold values (P < 0.05) indicate a significant difference in damage fraction between accessions.

**Vegetative stage**												
							***P*-value**					
	**Accession**	13	14	19	23	34	38	41	43	52	63	70
**V-statistic**	13		0.760	0.418	**0.006**	0.054	**0.008**	0.083	0.475	0.359	**0.006**	**0.006**
	14	31		0.838	0.014	0.154	**0.009**	0.760	0.103	0.541	**0.009**	**0.009**
	19	36	25		**0.008**	0.052	**0.006**	0.266	**0.041**	0.308	**0.006**	**0.006**
	23	55	52	54		0.541	0.103	0.185	**0.014**	0.414	**0.006**	**0.003**
	34	47	42	47	34		0.683	0.185	**0.019**	0.221	**0.008**	**0.008**
	38	54	45	55	44	32		0.053	**0.006**	**0.008**	**0.009**	0.123
	41	45	31	39	14	14	8		**0.041**	0.919	**0.006**	**0.019**
	43	20	11	7	3	4	0	7		**0.041**	**0.006**	**0.006**
	52	37	34	38	19	15	1	26	48		**0.006**	**0.019**
	63	55	45	55	55	54	45	55	55	55		0.944
	70	55	45	55	49	54	45	51	55	51	51	
**Generative stage**												
							***P*-value**					
	**Accession**	13	14	19	23	34	38	41	43	52	63	70
**V-statistic**	13		**0.025**	0.838	0.683	0.066	0.308	0.636	0.080	**0.006**	0.683	1.000
	14	5		**0.025**	**0.014**	0.760	0.262	**0.014**	0.760	0.221	**0.011**	**0.032**
	19	25	50		0.683	**0.025**	0.126	0.083	**0.032**	**0.008**	0.665	0.919
	23	23	52	23		**0.006**	0.262	0.308	0.066	**0.014**	0.126	0.475
	34	9	31	5	0		0.475	**0.014**	1.000	0.154	**0.006**	**0.019**
	38	17	39	12	16	35		**0.011**	0.415	0.066	**0.014**	0.052
	41	27	52	45	38	52	53		**0.024**	**0.008**	0.286	0.554
	43	5	31	6	9	27	19	3		0.221	**0.032**	**0.032**
	52	0	15	1	3	13	9	1	15		**0.008**	**0.011**
	63	32	53	46	43	55	52	32	49	54		0.286
	70	27	49	29	35	51	47	17	49	53	53	

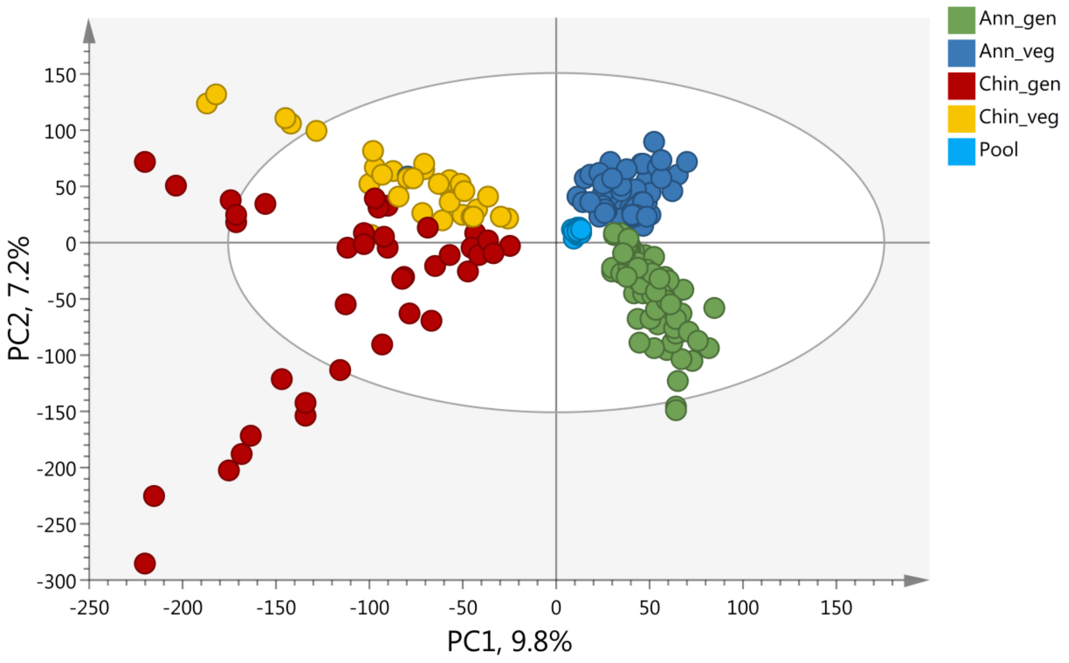

Figure S1. Principal Component Analysis (PCA) plot of the LC-MS positive ionization mode, all mass peaks of all samples, colors indicate species/developmental stage.

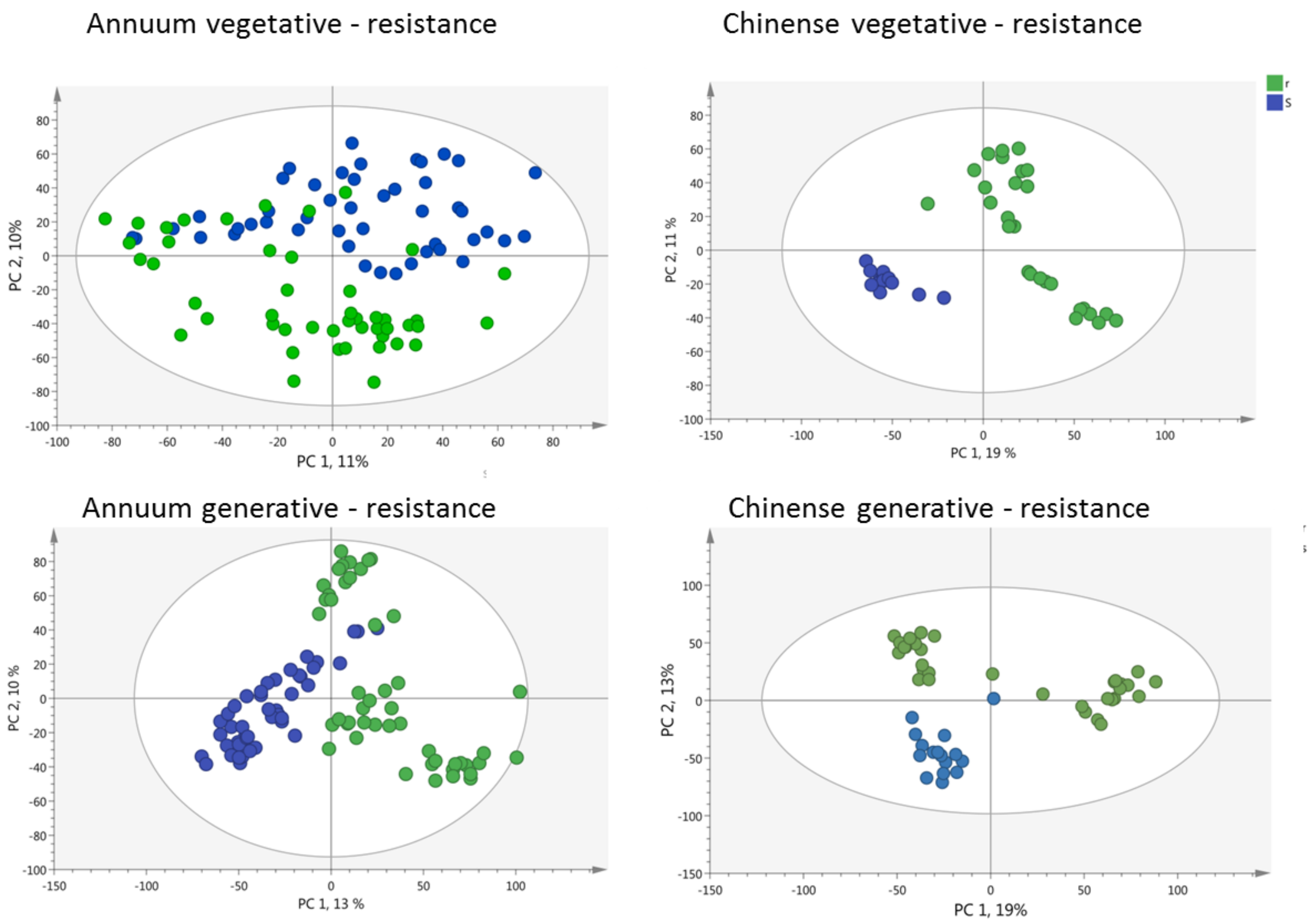

Figure S2. PCA plots per species/developmental stage of LCMS mass peaks, colors by thrips resistance or susceptibility of accessions, green is resistant, blue susceptible.

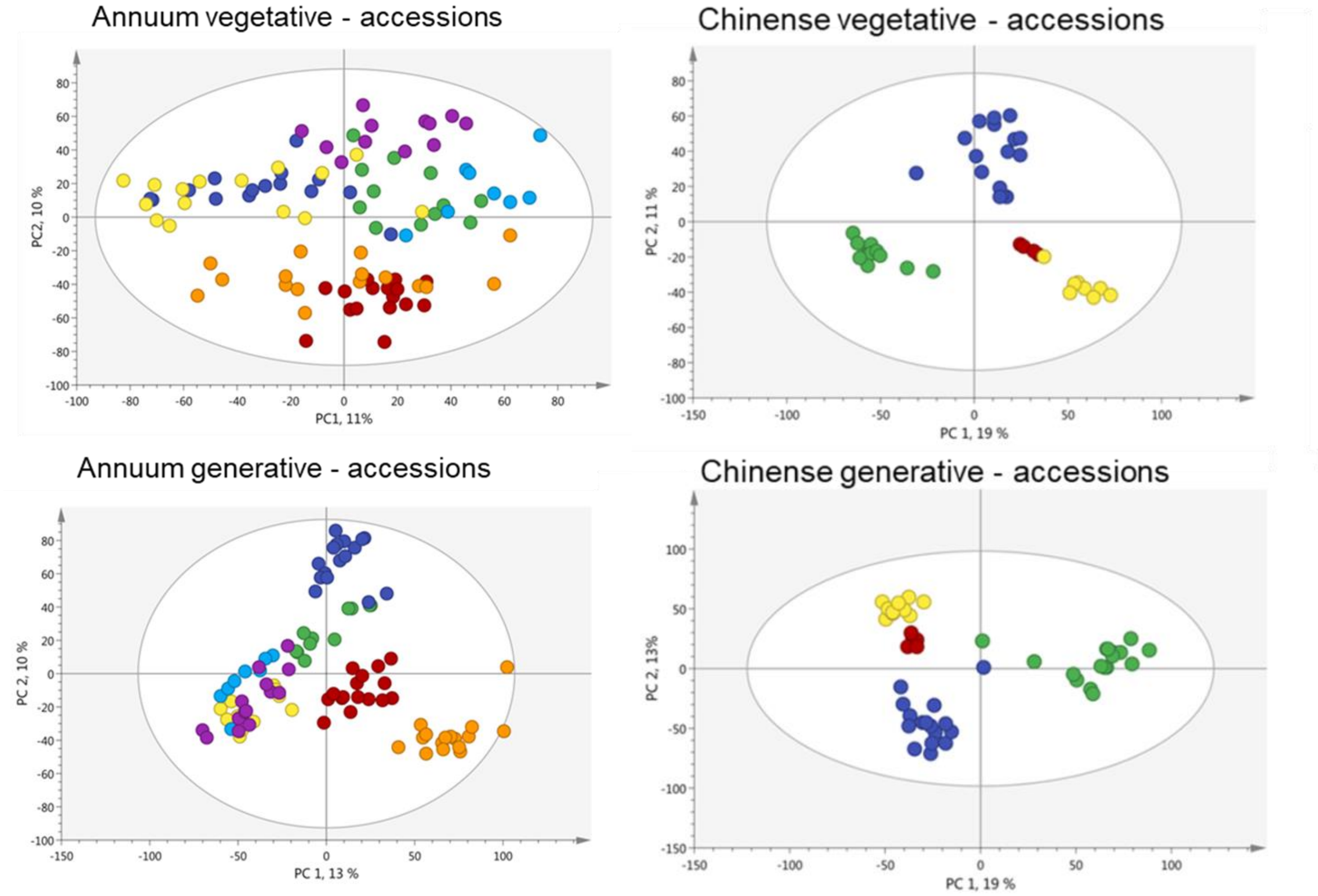

Figure S3. PCA plots per species/developmental stage of LCMS mass peaks, colors by accessions. *C. annuum* green: 14, blue: 19, red: 23, yellow: 34, light blue: 43, purple: 52, orange: 63. *C. chinense* green: 13, blue: 38, red: 41, yellow: 70.

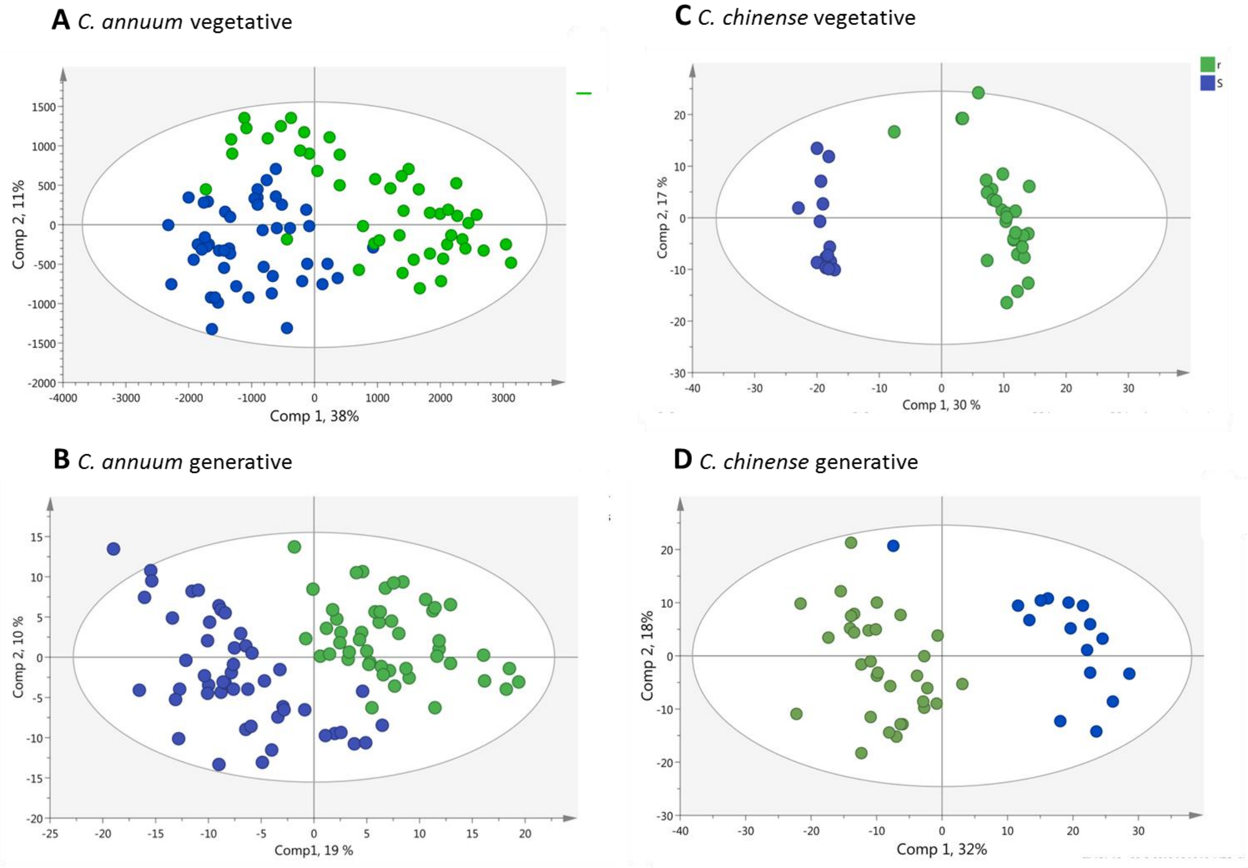

Figure S4. PLS-DA plots per species/developmental stage of LC-MS mass peak data of leaves of each *Capsicum* species and developmental stage. A) *C. annuum* vegetative stage, B) *C. annuum* generative stage, C) *C. chinense* vegetative stage, D) *C. chinense* generative stage. Green dots indicate resistant accessions, blue dots indicate susceptible accessions. All models were significant with P < 0.0001. N = 8-16 per accession.

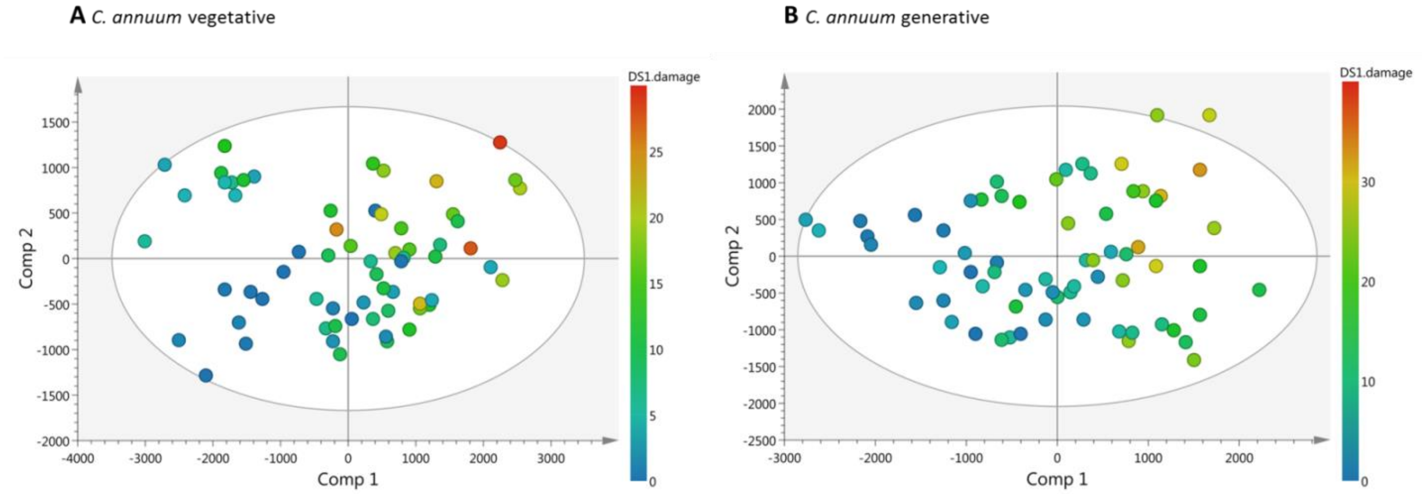

Figure S5. PLS regression plots of *Capsicum annuum* LC-MS mass peaks and thrips damage. A) vegetative stage, B) generative stage. Both models were significant P < 0.005. Models for *Capsicum chinense* were not significant (P > 0.7) and are not shown.

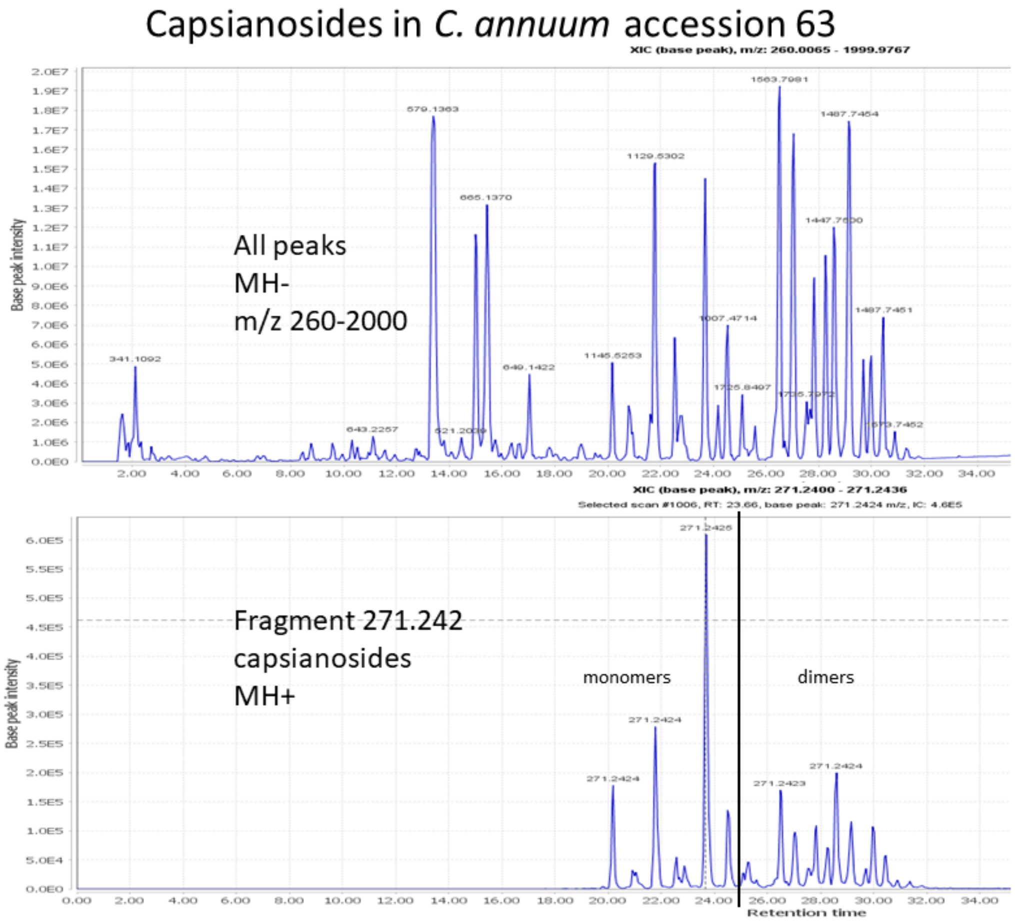

Fig. S7 Full metabolomic profile of *Capsicum annuum* CGN 63 (upper panel) in LC-MS negative ionisation mode; acyclic diterpene glycoside (capsianoside) signature fragment 271.242 (lower panel) of the same sample in LC-MS positive ionisation mode.
